# Potential Antifungal Activity of Retinoids Against Non-*albicans Candida* Species

**DOI:** 10.3390/microorganisms14040759

**Published:** 2026-03-27

**Authors:** Terenzio Cosio, Enrico Salvatore Pistoia, Francesca Pica, Augusto Orlandi, Elena Campione, Roberta Gaziano

**Affiliations:** 1 Department of Basic Biotechnological Sciences, Intensive and Perioperative Clinics, Università Cattolica del Sacro Cuore, L.go F. Vito 8, 00136 Rome, Italy; terenziocosio@gmail.com; 2 Department of Experimental Medicine, University of Rome Tor Vergata, 00133 Rome, Italy; pistoiae@uniroma2.it (E.S.P.); pica@uniroma2.it (F.P.); 3 Pathological Anatomy Institute, Department of Biomedicine and Prevention, University of Rome Tor Vergata, 00133 Rome, Italy; orlandi@uniroma2.it; 4 Dermatologic Unit, Department of Systems Medicine, University of Rome Tor Vergata, 00133 Rome, Italy; elena.campione@uniroma2.it

**Keywords:** retinoids, ATRA, trifarotene, tazarotene, *Candida auris*, *Candida* non-*albicans*, biofilm

## Abstract

Non-*albicans Candida* (NAC) species have emerged as significant opportunistic pathogens due to their reduced susceptibility to antifungal agents combined with their strong ability to form biofilms. The severity of systemic candidiasis caused by NAC species highlights the need for novel antifungal strategies. Retinoids, a group of compounds derived from vitamin A, have been demonstrated to possess significant antifungal activity against the reference strain *C. albicans* ATCC 2091. This study aimed to assess the antifungal potential of three retinoids, all-trans retinoic acid (ATRA), trifarotene, and tazarotene, against NAC clinical isolates. Various concentrations of the retinoids (from 1 mM to 0.06 mM) were tested in vitro against the growth, adhesion, and biofilm development of NAC species, including *Candida glabrata*, *Candida krusei*, and *Candida tropicalis*, as well as a reference strain of *C. auris* (CDC B11903). At 1 mM, all three compounds maximally inhibited the growth, adhesion, and biofilm formation of all tested NAC species. At lower concentrations (0.5–0.06 mM), *C. krusei* remained the most susceptible, especially to tazarotene. Tazarotene also showed a strong inhibitory effect on *C. auris* and *C. glabrata* at 0.5–0.25 mM; however, this effect was weaker than that observed against *C. krusei*. At low concentrations (0.12–0.06 mM), only trifarotene induced a mild but statistically significant inhibition of *C. tropicalis* growth. Trifarotene at 0.5 mM was also the most effective retinoid in inhibiting *C. glabrata* and *C. tropicalis* adherence and biofilm formation, with inhibitory activity maintained even at sub-0.5 mM concentrations (0.25–0.12 mM). Overall, the results suggest that all three retinoids exhibited dose-dependent and species-specific antifungal activity against NAC species, supporting their potential as novel, tailored antifungal agents against drug-resistant *Candida* strains.

## 1. Introduction

The incidence of fungal infections has steadily risen in recent decades, posing a significant challenge to global healthcare systems. While *C. albicans* has historically been considered the principal leading cause of candidiasis, NAC species have emerged as an important cause of healthcare-associated infections, especially in severely ill patients [1,2]. These species include *Candida glabrata (Nakaseomyces glabrata)*, *Candida krusei (Pichia kudriavzevii)*, *Candida tropicalis*, *Candida parapsilosis*, and the new emerging multidrug-resistant (MDR) *Candida auris (Candidozyma auris)*, which are considered high-priority pathogens according to the World Health Organization [3,4,5]. Like *C. albicans*, NAC species can colonize different areas of the body in healthy individuals as commensals, such as the oral cavity, gastrointestinal, and urogenital tracts, as well as cutaneous surfaces. In normal conditions, a delicate equilibrium exists among *Candida*, the host immune system, and local microbiota [6]. However, under some circumstances, such as weakening of the immune system, dysbiosis associated with the use of broad-spectrum antibiotics, or disruption of physical cutaneous/mucosal barriers, such as *Candida* spp., can overgrow and/or switch from yeast to filamentous form, leading to a variety of infections ranging from superficial muco-cutaneous to hospital-associated systemic life-threatening infections in severely immunocompromised patients [7]. The rising prevalence of NAC species is particularly concerning due to their virulence factors and resistance to the current antifungals. The ability of *Candida* yeasts to adhere and form biofilms on implanted medical devices represents one of the major virulence factors in the development of hospital-associated invasive candidiasis in critically ill patients [8,9]. In fact, within the biofilm, *Candida* cells are less susceptible to antifungal treatments. Further, the biofilm protects *Candida* from the host immune response and serves as a reservoir of *Candida* cells contributing to the spread of infection [10]. In addition to the ability to form biofilm, one of the most pressing challenges associated with NAC infections is their notable resistance to commonly used antifungal agents, including azoles, echinocandins, and polyenes [10,11,12]. This drug resistance phenomenon not only complicates treatment strategies but also contributes to increased morbidity and mortality rates [13,14]. The misuse and overuse of antifungals in clinical and agricultural settings have exacerbated this issue, facilitating the emergence of MDR strains. Notably, the increasing emergence of fluconazole- and echinocandin-resistant NAC is of particular concern due to the increased incidence of infections caused by NAC worldwide. New emerging NAC species such as *C. auris* can even exhibit resistance to multiple classes of existing antifungal agents [15,16]. So far, treatment options for invasive fungal infections are limited. Patients at highest risk for invasive candidiasis often have multiple comorbidities, including immunosuppression, which may limit the therapeutic efficacy of current antifungal therapies. This is especially true for therapies that rely on fungistatic drugs, even in the absence of drug resistance. Therefore, the limitations of existing therapeutic options underscore the urgent need for novel antifungal strategies to combat drug-resistant NAC species. In this context, retinoids, which are natural or synthetic derivatives of vitamin A, have been shown to exhibit significant antifungal activity [17,18,19]. In particular, we demonstrated for the first time the in vitro effectiveness of retinoid compounds, including all-trans retinoic acid (ATRA), trifarotene, and tazarotene, in inhibiting the growth, germination, and biofilm formation of the reference strain *Candida albicans* ATCC 2091 [18,19]. In a rat model of invasive pulmonary aspergillosis (IPA), ATRA reduced mortality similarly to posaconazole and enhanced macrophage phagocytosis of *Aspergillus* conidia in vitro [17].

Based on this evidence, the aim of the present study was to evaluate the antifungal potential of ATRA, trifarotene, and tazarotene against clinical isolates of NAC species, including *C. krusei*, *C. glabrata*, a fluconazole-resistant *C. tropicalis* isolate, and the reference strain *C. auris* CDC B11903.

## 2. Materials and Methods

### 2.1. Candida Strain and Culture Conditions

In all experiments performed in vitro, the standard *C. auris* strain CDC B11903 and clinical isolates of *C. tropicalis*, *C. glabrata*, and *C. krusei* (BioProject NIH, number PRJNA1170201) were used. The strains were cultured on Sabouraud dextrose agar (SDA; Difco Laboratories, Detroit, MI, USA), supplemented with chloramphenicol, for 24 h at 30 °C. After incubation, *Candida* cells were harvested by rinsing the slant culture with sterile saline, counted using a Bürker chamber (catalog No. BR719520, Sigma-Aldrich, Burlington, MA, USA), and standardized to the required concentration.

### 2.2. Antifungal Susceptibility Testing

The antifungal susceptibility testing of *C. auris*, *C. tropicalis, C. krusei*, and *C. glabrata* was performed with the SENSITITRE™ YEASTONE™ (SYO^®^, TREK Diagnostics Systems, Thermo-Fisher, Leicestershire, UK) panels, following the manufacturer’s protocol with slight modifications. Briefly, the inoculum for SYO^®^ (Thermo-Fisher, Leicestershire, UK) was prepared by picking four well-isolated colonies of at least 1 mm in diameter. *Candida* cells were emulsified in sterile saline and prepared to a turbidity equal to 0.5 McFarland standard. Additionally, 20 μL of *Candida* suspension was inoculated into the SYO^®^ broth, and the concentration of the working solution was standardized to 2 × 10^3^ CFU/mL using a Bürker chamber, following the Clinical and Laboratory Standards Institute (CLSI) M27M44S guidelines [20]. Then, 100 µL of the broth suspension was transferred into each well of the SYO^®^ panels and incubated without agitation at 35 °C for 24 h. After incubation, the plates were read visually under normal laboratory lighting. Visible yeast proliferation was indicated by a color change from blue (negative, signifying no fungal growth) to pink (positive, indicating fungal growth). A range of antifungal agents was assessed, including fluconazole, voriconazole, itraconazole, isavuconazole, posaconazole, caspofungin, micafungin, anidulafungin, and amphotericin B. Data were reported as geometric means (GM) based on two independent experiments each conducted in duplicate.

### 2.3. Antimicrobial Compounds

Stock solutions of ATRA (catalog No. R2625; Sigma-Aldrich, Burlington, MA, USA), trifarotene (catalog No. AMBH93D58E72; Merck Life Science, Darmstadt, Germany), tazarotene (catalog No. T7080; Merck Life Science), and amphotericin B (AmB) (catalog No. 1397-89-3; analytical-grade powder; Sigma-Aldrich) were prepared by dissolving each compound in 50% dimethyl sulfoxide (DMSO; Sigma-Aldrich). These solutions were subsequently diluted in RPMI 1640 culture medium to achieve a final DMSO concentration of 2.5% DMSO (*v*/*v*). RPMI 1640 containing 2.5% DMSO served as the negative control in experiments.

### 2.4. Cell Growth Rate

To evaluate the antifungal potential of ATRA, trifarotene, and tazarotene on NAC species growth, 2 × 10^6^ *Candida* cells were seeded into 96-well flat-bottom plates (Thermo Scientific™ Nunc™ MicroWell™ 96-Well, Nunclon Delta-Treated, Flat-Bottom Microplate; Waltham, MA, USA) containing 100 μL of RPMI 1640 medium supplemented with 10% fetal calf serum (FCS; catalog No. 9014-81-7; Sigma-Aldrich) either in the absence or presence of the test compound. Various concentrations of retinoids were tested, ranging from 1 mM to 0.06 mM, corresponding to trifarotene 459.6–28.72 µg/mL, tazarotene 351.5–21.96 µg/mL, and ATRA 300–18.75 µg/mL. AmB was included as a positive control, at concentrations of 2.2–0.14µM (2–0.12 μg/mL), as established in our previous studies [18,19]. Each well received 100 μL of the respective compound. Controls included wells containing only *Candida* cells in 200 μL of culture medium (positive control) and wells with 200 μL of culture medium alone or 100 μL of medium plus 100 μL of each retinoid or AmB without *Candida* (negative controls). The plates were incubated at 30 °C for 24 h. Following incubation, cell growth was quantified by measuring absorbance at 510 nm, using an enzyme-linked immuno-sorbent assay (ELISA) reader (ThunderBolt^®^, Tecan s.r.l., Milan, Italy). The initial concentrations of *C. auris*, *C. tropicalis*, *C. glabrata*, and *C. krusei* were considered as the concentration at 0 time. Inhibitory concentrations (IC)_50_ and IC_90_ values for ATRA, trifarotene, and tazarotene were estimated from growth inhibition dose–response data by non-linear regression. For each experiment, technical triplicates were averaged per concentration and fitted on log10-transformed concentrations using a four-parameter logistic model with variable slope (Hill equation):

Y=Bottom+(Top−Bottom)/(1+10^((LogIC_{50}−X)×HillSlope))

where Y is the percent inhibition and X is log10 (concentration).

IC_50_ and IC_90_ were interpolated from the fitted curves and reported with 95% confidence intervals [21,22].

### 2.5. Adhesion Assay on Abiotic Surface

The adhesion capacity of *Candida* spp. to abiotic surfaces was assessed using the XTT [2,3-bis(2-methoxy-4-nitro-5-sulfophenyl)-2H-tetrazolium-5-carboxanilide] reduction assay, as originally described by Hawser and Douglas [23]. Clinical isolates of *C. tropicalis*, *C. glabrata*, *C. krusei*, and the reference strain of *C. auris* (CDC B11903) were grown overnight in YPD at 30 °C. Cells were collected by centrifugation, washed twice with PBS, and resuspended in RPMI 1640 medium to a final density of 1 × 10^6^ cells/mL. A volume of 100 µL from each cell suspension was added to flat-bottom 96-well polystyrene microtiter plates and incubated at 37 °C for 90 min to permit cell attachment. Non-adherent cells were discarded by gently washing the wells twice using sterile PBS. After adhesion, cells were treated with ATRA, trifarotene, or tazarotene at concentrations ranging from 1.00 to 0.06 mM. The plates were then incubated with 100 µL of XTT–menadione solution (0.5 mg/mL XTT with 1 µM menadione) at 37 °C for 2 h in the dark under gentle orbital shaking (80–100 rpm) to promote reagent diffusion. Measurements of absorbance at 490 nm were performed using a microplate reader. Values represent the mean ± SD of three separate experiments, each carried out in triplicate.

### 2.6. Quantification of Candida Biofilm by Crystal Violet and XTT Assays

The effects of retinoids on NAC species biofilm production was evaluated by measuring total biomass and metabolic activity. Briefly, a total of 2 × 10^5^ *Candida* cells were seeded into 96-well flat-bottom plates containing 200 μL of RPMI 1640 medium, supplemented with 10% FCS and incubated at 37 °C for 24 h either without treatment or with various concentrations of the tested compounds or AmB. Biofilm biomass was quantified by crystal violet (CV) staining as previously described [18,19].

Following incubation, each well was carefully rinsed twice with 200 μL of phosphate-buffered saline (PBS) to eliminate non-adherent cells and then allowed to air-dry for 20 min at 35 °C. The attached biofilms were stained with 150 μL of 0.4% aqueous CV solution for 20 min. Excess dye was then removed by washing the wells three times with distilled water. The bound dye was solubilized with 200 μL of 33% glacial acetic acid, and after 15 min, 100 μL of the resulting solution was transferred to a new microtiter plate [18,19]. To evaluate the impact of retinoids on pre-formed NAC biofilm, 24 h old biofilms were treated with the tested concentrations (0.06 mM–1.0 mM) and incubated for an additional 24 h.

Biofilm metabolic activity was evaluated using the XTT reduction assay. Fresh solutions of XTT [2,3-bis(2-methoxy-4-nitro-5-sulfophenyl)-2H-tetrazolium-5-carboxanilide sodium salt] and menadione (Sigma-Aldrich, Milan, Italy) were prepared and mixed at a 20:1 (*v*/*v*) ratio. For the assay, 42 μL of the XTT–menadione mixture and 158 μL of PBS were added to each well containing pre-washed biofilms. Plates were incubated at 37 °C for 3 h in the dark. After incubation, 100 μL of the colored supernatant was transferred to a fresh microtiter plate, and absorbance was measured at 490 nm.

Experiments were performed in triplicate across three independent runs. The results are expressed as mean absorbance ± SD, with values corrected by subtracting the background from negative control wells (without cells).

After CV staining, the biofilms were analyzed using a light microscope (Olympus, Carl Zeiss, Singapore) with 40× magnification objective lenses to observe biofilm morphology and evaluate hyphal development. Images were recorded using the accompanying digital camera.

### 2.7. Assessment of Fungal Viability and Recovery After Retinoid Exposure

For viability assessment, *Candida* cells were exposed for up to 7 days to the tested concentrations (0.06–1.0 mM) of ATRA, tazarotene, or trifarotene under standard incubation conditions. Following exposure, yeast cells were collected by centrifugation (3000× *g*, 5 min), washed twice with PBS to remove residual compound, and resuspended in fresh RPMI 1640 medium supplemented with 10% FCS in the absence of retinoids. Aliquots of the washed suspensions were seeded in flat-bottom 96-well polystyrene plates (2 × 10^5^ cells/well in 200 µL final volume) and incubated at 37 °C for up to 48 h. The primary endpoint was the CFU log reduction; immediately after wash-out (t = 0) and at 24 h post-wash, cell suspensions were serially diluted, plated on SDA, and incubated at 30 °C for 24–48 h to determine CFU. A reduction ≥3-log_10_ CFU compared to untreated controls was defined as fungicidal, while reductions <3-log_10_ with regrowth at 24–48 h were interpreted as fungistatic [24,25]. The secondary endpoint was the regrowth kinetics; optical density (OD_490_) was measured every 3 h for up to 24–48 h in drug-free medium. AUC_0–24_h was calculated, and a Recovery Index (RI%) was defined as:

$$RI\%=100\times \frac{AUC_{post\text{-}wash}}{AUC_{control}}$$

Values <20% indicated strong residual inhibition, 20–60% partial recovery, and >60% near-complete recovery [24,25].

Microscopic examination at 24 h post-wash was performed to document species-appropriate growth, and these observations were used to confirm the growth of each strain by the investigator.

### 2.8. Statistical Analysis

Data on the growth, biofilm formation, and metabolic activity of *Candida* spp. were analyzed using GraphPad Prism 10.2.0. Statistical significance between tested compounds and controls was assessed with two-way ANOVA or chi-square tests, followed by Dunnett’s multiple comparisons test when each treatment was compared with the control, or Tukey’s multiple-comparisons test when all pairwise comparisons were performed. The results are expressed as the means ± SD from three independent experiments carried out in triplicate. The significance level for *p* values was considered as * *p* < 0.05; ** *p* < 0.01; *** *p* < 0.001; and **** *p* < 0.0001.

## 3. Results

### 3.1. Antifungal Susceptibility Profile of Non-albicans Candida Species

First, we assessed the antifungal sensitivity of NAC species, including *C. tropicalis*, *C. glabrata*, and *C. krusei* isolates, as well as *C. auris* reference strain CDC B11903, using the Sensitrite™ YeastOne™ (SYO) system. The MIC values (μg/mL) of NAC to the nine antifungals commonly used in the clinic are shown in Table 1. The data demonstrates full susceptibility of *C. glabrata* and *C. auris* to all antifungal agents tested. In contrast, *C. krusei* exhibited intrinsic resistance to fluconazole, as expected, while *C. tropicalis* showed reduced susceptibility to fluconazole, with both species presenting MIC values exceeding 4 μg/mL, indicating potential resistance or tolerance, according to CLSI [26].

### 3.2. Growth Inhibitory Activity of Retinoids on Non-albicans Candida Species

**Figure 1:**
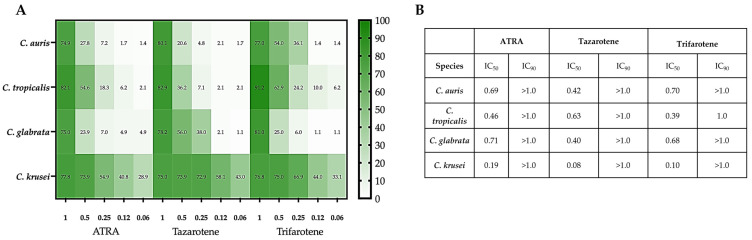
Retinoid-mediated inhibition of planktonic growth in non-*albicans Candida*. Planktonic *Candida* cells were incubated for 24 h at 30 °C in the absence or presence of ATRA, tazarotene, or trifarotene (1.00–0.06 mM). (**A**) Heatmap showing the mean percentage growth inhibition vs. untreated control for *Candida auris* CDC B11903 and clinical isolates of *C. tropicalis*, *C. glabrata*, and *C. krusei*; color intensity reflects inhibition (0–100%). (**B**) Table reporting IC50 and IC90 (mM) for each retinoid–species combination, estimated from dose–response data by non-linear regression (four-parameter logistic model). Data derives from three independent experiments, each performed in technical triplicate. Corresponding bar plots with SD and statistical significance are provided in Figure S1. Amphotericin B was included as a positive control (Figure S2).

We demonstrated previously that retinoids, and specifically ATRA, trifarotene, and tazarotene, induced a strong inhibitory activity against the growth and biofilm formation of a reference strain of *C. albicans* (ATCC 2091) [18,19]. Based on this evidence, in the present study, we aimed to evaluate the antifungal potential of these compounds in inhibiting the growth of planktonic cells of some clinical isolates of NAC species, i.e., *C. krusei*, *C. glabrata*, and *C. tropicalis*. A reference strain of *C. auris* (CDC B11903) was also included in this study. Figure 1A shows that NAC species exhibited significant diversity in sensitivity to the individual retinoids. Notably, all three retinoids at 1 mM exerted the highest inhibitory activity against the selected NAC strains (Figure S1, one-way ANOVA; *p* < 0.001). At 0.5 mM, the tested compounds showed the strongest antifungal activity against *C. krusei*, comparable to the activity observed at 1 mM for the other *Candida* species. Interestingly, the highest inhibition against *C. krusei* was observed even after exposure to retinoids at concentrations below 0.5 mM (ranging from 0.25 mM to 0.06 mM), with tazarotene demonstrating the greatest efficacy among the tested compounds. Moreover, 0.5 mM tazarotene significantly inhibited the growth of *C. auris* and *C. glabrata*, with inhibition rates of 55.5% and 56%, respectively (one-way ANOVA; *p* < 0.01 and *p* < 0.001). Its efficacy was found to be superior to that of ATRA (29% and 24% inhibition, respectively) and trifarotene (21% and 25% inhibition, respectively; two-way ANOVA; *p* < 0.001). Even at a lower concentration (0.25 mM), tazarotene retained antifungal activity against both species, though less effectively than against *C. krusei*.

Compound concentrations between 0.12 mM and 0.06 mM showed no inhibitory activity against *C. auris* and *C. glabrata* except for trifarotene, which induced a slight yet statistically significant inhibition of the growth of *C. tropicalis* (one-way ANOVA; *p* < 0.05).

Based on concentration–response curves from the growth inhibition assay (0.06–1.0 mM), IC_50_ and IC_90_ values were estimated for each compound and species (Figure 1B). Overall, inhibitory activity was species-dependent, with *C. krusei* showing the highest susceptibility, particularly to tazarotene (IC_50_ 0.08 mM), followed by trifarotene (IC_50_ 0.10 mM) and ATRA (IC_50_ 0.19 mM). In contrast, *C. glabrata* and *C. auris* displayed higher IC_50_ values, with tazarotene being the most active compound against *C. glabrata* (IC_50_ 0.40 mM). In *C. tropicalis*, trifarotene achieved the highest maximal inhibition, although all the tested retinoids reached IC_90_ only at concentrations above 1 mM, consistent with a predominantly fungistatic effect.

### 3.3. Candida Growth Recovery Following Retinoid Exposure

**Figure 2:**
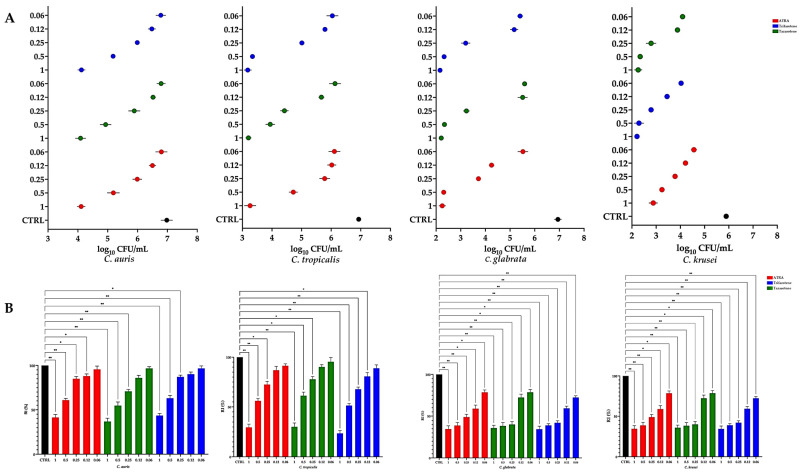
Recovery of *Candida* growth inhibition after retinoid treatment. *Candida* strains were exposed to retinoids (0.06–1.00 mM) for 7 days and then washed and resuspended in drug-free medium. (**A**) CFU counts (log_10_ scale) at t = 0 and 24 h post-wash. A ≥ 3-log_10_ reduction was used as the fungicidal threshold. (**B**) Recovery Index (RI) calculated from the AUC_0–24_h of growth curves expressed as a percentage of the untreated control and showed a dose- and species-dependent pattern of recovery, with *C. krusei* being the most susceptible. Data are means ± SD (error bars) from three independent experiments. Statistical significance was determined by two-way ANOVA with Tukey’s post hoc test, * *p* < 0.05; ** *p* < 0.01.

Following 7-day exposure to ATRA, tazarotene, or trifarotene, *Candida* cells were washed and transferred to drug-free SDA to evaluate residual killing and post-exposure recovery (Figure 2). At 24 h post-washout, *C. auris* did not reach the ≥3-log_10_ fungicidal threshold at any tested concentration, although CFU recovery was reduced at 1.0 mM. In contrast, a ≥3-log_10_ reduction in CFU relative to the untreated control was observed at 1.0 mM for *C. tropicalis*, *C. glabrata*, and *C. krusei*, indicating a concentration-dependent residual effect. Despite this, viable colonies were still recovered under all conditions, consistent with incomplete killing and suggesting predominantly fungistatic activity at sub-millimolar concentrations, with only partial fungicidal effects at the highest dose.

Regrowth kinetics in drug-free medium, quantified by the Recovery Index, further supported species- and compound-dependent post-washout effects. *C. auris* recovered comparatively well at lower concentrations, with RI approaching control values at 0.25–0.06 mM, while RI decreased at 1.0 mM. *C. tropicalis* showed a more pronounced and dose-dependent suppression of recovery, with trifarotene yielding the lowest RI at higher concentrations compared with the other retinoids. *C. glabrata* and *C. krusei* exhibited sustained post-exposure growth impairment, with low RI values persisting across 1.0–0.25 mM for all compounds and recovery improving mainly at the lowest concentrations. Overall, wash-out experiments indicate a strong concentration effect on residual growth suppression, with marked inter-species variability and limited evidence of sterilising activity.

### 3.4. Retinoids Inhibit Cell Adhesion

**Figure 3:**
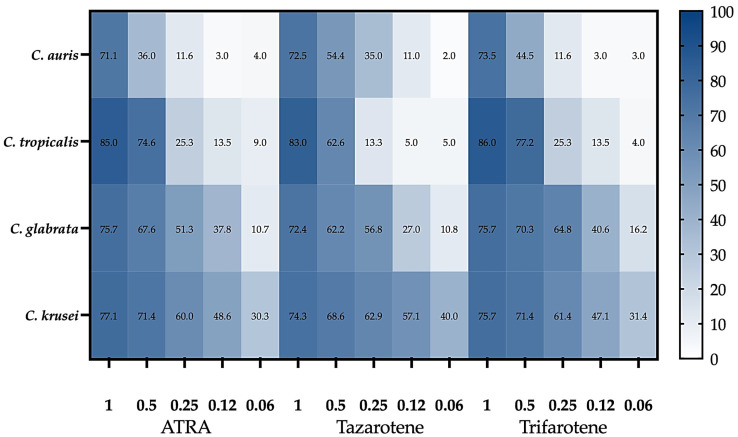
Retinoid-mediated inhibition of *Candida* adhesion. Planktonic *Candida* cells were incubated for 24 h at 30 °C in the absence (control) or presence of ATRA, tazarotene, or trifarotene (1.00–0.06 mM). The heatmap reports the mean percentage inhibition of adhesion vs. untreated control for *Candida auris* CDC B11903 and clinical isolates of *C. tropicalis*, *C. glabrata*, and *C. krusei*; color intensity reflects the magnitude of inhibition (0–100%). Data are from three independent experiments, each performed in technical triplicate. Corresponding bar plots with SD and statistical significance are provided in Figure S3.

The impact of retinoids on the adhesion capacity of NAC species to abiotic surfaces was evaluated using the XTT reduction assay. Consistent with the observed growth inhibition, Figure 3 shows that at 1 mM, all retinoids significantly impaired adhesion of all tested NAC species (two-way ANOVA, *p* < 0.001). Among the three tested compounds, trifarotene exhibited the strongest inhibitory effect on *C. tropicalis* adhesion at 1 mM. In contrast, tazarotene showed superior anti-adhesion activity against *C. krusei*, even at lower concentrations ranging from 0.25 mM to 0.06 mM (One-way ANOVA, *p* < 0.01).

Tazarotene also showed the highest inhibition of *C. auris* adhesion at concentrations between 0.5 mM and 0.12 mM, although it was less effective than against *C. krusei*. In contrast, trifarotene at concentrations of 0.5–0.12 mM was the most effective compound against both *C. glabrata* and *C. tropicalis*, with efficacy against *C. glabrata* also observed at 0.06 mM (one-way ANOVA, *p* < 0.05).

### 3.5. Retinoids Prevent the Hyphal Growth in C. tropicalis

**Figure 4:**
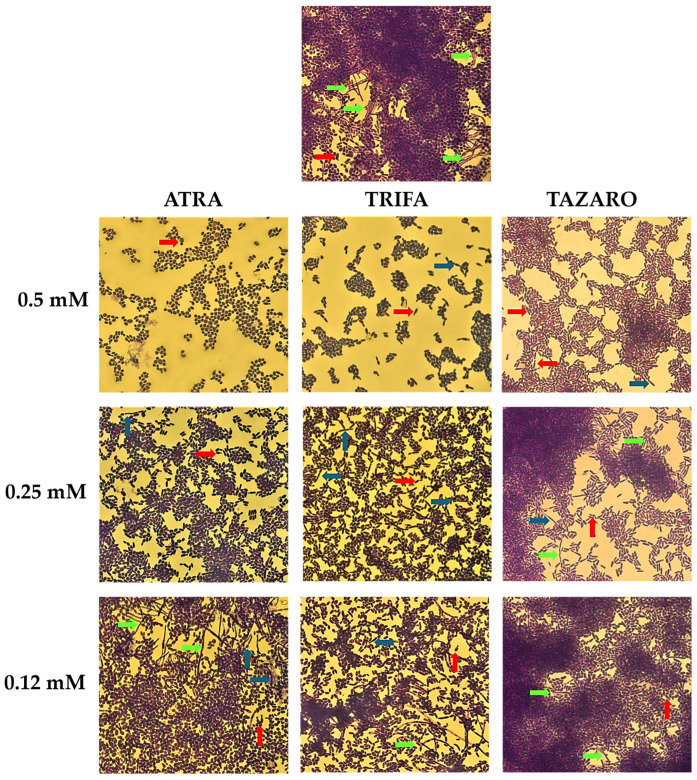
Morphological analysis of *Candida tropicalis* cultures after exposure to retinoids. Representative images of *Candida* cells stained with crystal violet and observed under an optical microscope with 40× magnification objective lenses. The staining highlights cellular morphology, allowing visualization of yeasts (red arrow) and filamentous forms, such as pseudohyphae (blue arrow) and true hyphae (green arrow).

Among the non-*albicans* species, *Candida tropicalis* stands out for its pronounced ability to undergo morphological transition toward true hyphal forms, a trait less evident or entirely absent in other *Candida* species, such as *C. auris*, *C. glabrata*, and *C. krusei*, which exhibit more limited filamentous growth. The microscopic analysis of *C. tropicalis* cultures in Figure 4 shows that ATRA, at concentrations ranging from 0.5 mM to 0.25 mM, exerted a clear inhibitory effect on hyphal growth, while at 0.12 mM, it was ineffective in preventing the morphological switching. In contrast, neither trifarotene nor tazarotene was able to arrest the filamentous transition in *C. tropicalis*, as indicated by the presence of pseudohyphae or true hyphae after exposure to the two compounds at concentrations of 0.5 mM and 0.25 mM.

### 3.6. Anti-Biofilm Effects of Retinoids on Non-albicans Candida Species

**Figure 5:**
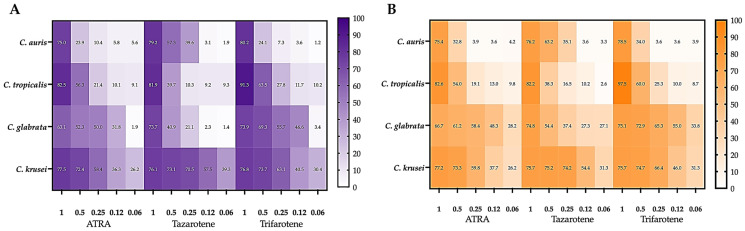
Retinoid-mediated inhibition of non-*albicans Candida* biofilms. *Candida* strains were allowed to form biofilms for 24 h at 30 °C in the absence (control) or presence of ATRA, tazarotene, or trifarotene (1.00–0.06 mM). Heatmaps report the mean percentage inhibition vs. untreated control for (**A**) biofilm biomass and (**B**) metabolic activity for *Candida auris* CDC B11903 and clinical isolates of *C. tropicalis*, *C. glabrata*, and *C. krusei*; color intensity reflects inhibition (0–100%). Data from three independent experiments, each performed in technical triplicate, are shown. Bar plots with SD and statistical significance are shown in Figures S4 and S5. Amphotericin B was included as a positive control (Figures S6 and S7).

Next, the impact of retinoids on the biofilm biomass and metabolic activity of NAC species was evaluated. As shown in Figure 5, consistent with the inhibitory pattern observed for *Candida* growth and adhesion, at 1 mM, all retinoids demonstrated maximal inhibitory effects against the biofilms of all tested NAC species (*p* < 0.001, two-way ANOVA), with trifarotene being the most effective against *C. tropicalis* (*p* < 0.05, two-way ANOVA). At 0.5 mM, ATRA, trifarotene, and tazarotene maintained strong anti-biofilm activity against *C. krusei*, comparable to their efficacy at 1 mM. Notably, *C. krusei* was the most susceptible species, with significant inhibition observed even at 0.25–0.06 mM, particularly with tazarotene. Tazarotene also showed superior anti-biofilm activity against *C. auris* at concentrations of 0.5–0.25 mM, compared to ATRA and trifarotene, although its efficacy was lower than that observed against *C. krusei*. At 0.5 mM, trifarotene displayed the strongest anti-biofilm activity against *C. glabrata* and *C. tropicalis*, albeit with reduced potency compared to its effect on *C. krusei.* Notably, trifarotene significantly inhibited *C. glabrata* biofilms even at sub-0.5 mM concentrations (0.25–0.12 mM), highlighting its potential as an effective agent against this species at lower doses.

By contrast, no effects were observed in vitro on pre-formed mature biofilms, even at high doses (1 mM) of any of the three retinoids, consistent with previous findings for ATRA against *C. albicans* ATCC 2091 [18].

## 4. Discussion

*C. albicans* has traditionally been recognized as the principal fungal pathogen in human disease. However, in recent years, increasing attention has turned toward NAC species, such as *C. auris*, *C. glabrata*, *C. krusei*, and *C. tropicalis*, due to their growing clinical relevance and increased resistance to conventional antifungal therapies [28,29]. Unlike *C. albicans*, which exhibits strong hyphal development, many NAC species display distinct morphological and pathogenic profiles. For example, *C. glabrata* predominantly exists only in the yeast form, lacking true hyphal structures; however, it is a significant cause of invasive candidiasis in healthcare settings, posing a therapeutic challenge due to its reduced susceptibility to azoles and the increasing emergence of echinocandin resistance [30]. *C. krusei* and *C. tropicalis* show varying degrees of filamentation, with *C. tropicalis* demonstrating greater virulence and filamentous growth under specific environmental conditions [31,32]. Both *C. tropicalis* and *C. krusei* are clinically relevant fungal species associated with hospital-acquired infections, particularly in immunocompromised patients. *C. krusei* exhibits intrinsic resistance to fluconazole, and the increasing resistance to echinocandins further limits therapeutic options, potentially impacting empiric treatment strategies in hospital settings [33]. Furthermore, among NAC species, the emerging *C. auris* is of particular concern due to its resistance to multiple classes of antifungal agents, including azoles, echinocandins, and polyenes, as well as environmental persistence and high potential for nosocomial outbreaks. In fact, unlike other *Candida* species, *C. auris* can survive for long periods on surfaces and medical equipment, facilitating transmission in hospital settings [34].

In addition to drug resistance, biofilm formation on medical devices by NAC species further contributes to their pathogenicity and treatment failure. In this regard, *C. auris* forms dense, drug-resistant biofilms that can withstand disinfection and contribute to persistent outbreaks in healthcare settings. Similarly, biofilms formed by *C. glabrata* and *C. tropicalis* are associated with increased resistance to azoles and echinocandins, thus complicating treatment strategies [35,36]. It is estimated that biofilm-associated infections account for over 80% of all microbial infections, posing a major challenge in both community and healthcare settings [9]. Once established on implanted medical devices or host tissues, *Candida* biofilms may act as reservoirs for persistent, drug-resistant fungal cells, leading to recurrent infections and systemic dissemination, including candidemia. Given the recalcitrant nature of *Candida* biofilms to conventional antifungal therapies, especially azoles, coupled with the rising incidence of drug resistance among *Candida* species, there is a critical need for the development of novel and effective antifungal treatments.

In this scenario, retinoids, a class of natural and synthetic derivatives of vitamin A, have emerged as promising candidates due to their strong antifungal activity observed in vitro, in vivo, and in clinical studies [17,18,19]. In our previous work, aimed at evaluating the potential antifungal efficacy of various retinoids, i.e., ATRA, trifarotene, and tazarotene, against the reference strain *C. albicans* ATCC 2091, ATRA demonstrated the strongest antifungal effect by inhibiting *C. albicans* growth, germination, and biofilm formation by targeting key regulatory proteins, such as the heat shock protein 90 (Hsp90) and the enzyme lanosterol 14α-demethylase (CYP51), thereby disrupting fungal stress responses and ergosterol biosynthesis [19]. However, these studies have been performed using the laboratory reference strains of *C. albicans* ATCC 2091, which is known to be fluconazole-sensitive, and information regarding their activity against NAC species remains unexplored to date.

In the present study, we investigated the potential effect of these retinoids on the growth, adhesion, and biofilm formation of clinical isolates of NAC species, including *C. glabrata*, *C. krusei*, and *C. tropicalis*, as well as a reference strain of *C. auris* (CDC B11903). The findings indicate differential antifungal activity of the tested retinoids against various non-*albicans Candida* species. Notably, at a concentration of 1 mM, all three compounds displayed maximal inhibitory activity against the growth, adhesion, and biofilm formation of all NAC tested, with trifarotene showing the strongest anti-biofilm and anti-adherence activities against *C. tropicalis*. All the compounds at 0.5 mM exerted marked antifungal, anti-adherence, and anti-biofilm effects against *C. krusei*, which remained highly susceptible even at lower concentrations (0.25–0.06 mM), particularly to tazarotene. At concentrations between 0.5 and 0.25 mM, tazarotene also exhibited superior antifungal activity by inhibiting the growth of *C. auris*, though its efficacy was lower than that observed against *C. krusei*. At lower concentrations (0.12 and 0.06 mM), none of the compounds inhibited the growth of *C. auris* or *C. glabrata*, whereas trifarotene produced a mild but statistically significant inhibition of *C. tropicalis*. Interestingly, colonies could still be recovered after washout even following 7-day exposure at 0.5–1.0 mM, supporting a predominantly fungistatic effect at sub-millimolar concentrations. However, at 1.0 mM, the reduction in CFU met the ≥3-log10 fungicidal threshold in *C. tropicalis*, *C. glabrata*, and *C. krusei*, whereas *C. auris* did not reach this threshold, indicating a dose- and species-dependent fungicidal component that emerges only at the highest concentration while a viable subpopulation persists. These results are consistent with our previous studies, demonstrating that treatment with 1.0 mM trifarotene or tazarotene led to 55% and 40% PI-positive (non-viable) cells, respectively, compared with 60% for ATRA in *C. albicans* ATCC 2091 [19], indicating fungicidal activity at this concentration.

At higher concentrations (0.5–0.25 mM), tazarotene was also the most effective agent against *C. auris*, significantly impairing both biofilm formation and adhesion. Conversely, trifarotene at 0.5 mM showed the greatest anti-biofilm and anti-adherence effects against *C. glabrata* and *C. tropicalis*. Notably, the inhibitory effects in *C. glabrata* were retained even at sub-0.5 mM concentrations (0.25–0.12 mM).

Overall, these findings suggest that the anti-adhesive property of retinoids is closely associated with their ability to inhibit biofilm formation in the NAC species tested. Since adhesion represents the initial and critical step in the process of biofilm formation, it is likely that the anti-biofilm activity of retinoids is driven, at least in part, by their anti-adhesive properties against *Candida* species. Future studies will aim to elucidate the mechanisms underlying the anti-adhesive effects of these compounds, with a particular focus on their potential to modulate the expression of key adhesin genes, such as those of the Agglutinin-Like Sequence (ALS) family and hyphal wall protein (HWP) 1, in *Candida* species. Like *C. albicans*, NAC species express adhesin proteins that facilitate host attachment and biofilm formation. However, their adhesin gene families often show significant differences in sequence, structure, and level of expression compared to those in *C. albicans*, reflecting species-specific adaptations to different host niches and pathogenic mechanisms [37,38].

Moreover, the results highlight that retinoids exert significant dose-dependent and species-specific antifungal effects against NAC strains. The observed variability in the responses of NAC species to distinct retinoid compounds in terms of growth, adhesion, and biofilm inhibition is not unexpected and likely reflects underlying interspecies variation in genetic composition, cell wall architecture, and regulatory pathways governing morphogenesis and drug susceptibility. Such findings are consistent with reports highlighting the heterogeneity of NAC species in their responses to conventional antifungal drugs [39,40]. Therefore, the demonstration that retinoids retain antifungal activity against structurally and genetically distinct *Candida* species, including drug-resistant clinical isolates, provides new evidence supporting their potential role as antifungal agents.

Moreover, the well-established safety profile of retinoids due to extensive use in dermatology and oncology [41] supports their potential therapeutic application against both sensitive and drug-resistant strains. Their antifungal effectiveness against fluconazole-resistant clinical isolates, such as *C. tropicalis* and *C. krusei*, further highlights their possible relevance in addressing emerging antifungal resistance. Importantly, the distinct susceptibility profiles in NAC species in this study suggest that retinoids may serve as promising adjunct or alternative anti-fungal agents, particularly when tailored to the specific *Candida* species. Although further investigation is required to elucidate the precise mechanisms driving these species-dependent effects, our results provide a basis for exploring retinoids as candidates for species-tailored antifungal therapies.

Although the concentrations required to achieve the strongest antifungal effects in our in vitro model may appear high, their translational relevance should be interpreted in light of the formulation-dependent pharmacology of retinoids. In conventional formulations, these compounds are constrained by poor water solubility, chemical and photochemical instability, and irritation, all of which may limit free-drug exposure and tolerability [42,43,44]. However, in future years, nanotechnology-based drug delivery systems, such as liposomes, polymeric nanoparticles, or solid-lipid nanoparticles, may allow us to achieve targeted deposition of the active agent at the sites of fungal infection, enhancing local drug bioavailability while minimizing systemic exposure [45,46]. Such formulations could, in principle, deliver retinoids directly into *Candida* biofilms, thereby ensuring a rapid therapeutic response, minimizing adverse effects. Rigorous optimization of carrier composition and release kinetics, alongside comprehensive safety and efficacy studies in relevant preclinical models, will be essential before nanocarrier-mediated retinoid delivery can be considered a viable clinical option for combating NAC species biofilm-associated infections [47]. In fact, in this study, the experiments conducted under in vitro conditions may not fully reflect the complexity of pharmacokinetics in vivo. Moreover, it is important to note that retinoids have already shown in vivo efficacy at lower concentrations in the treatment of onychomycosis, including cases involving *Candida* spp. [48,49]. In fact, this in vitro study should be regarded as a proof of concept; the experiments conducted under in vitro conditions may not fully reflect the complexity of pharmacokinetics in vivo. This apparent discrepancy further highlights that in vitro potency does not always directly predict in vivo activity, where additional factors such as tissue distribution, local accumulation, host microenvironment, keratin affinity, and modulation of fungal virulence may contribute to therapeutic efficacy. Other limitations of this study should be considered. First, the sample size of *Candida* isolates was limited, and although they are representative of several clinically relevant species, broader strain diversity could influence susceptibility patterns to retinoids. Second, this study did not investigate the molecular mechanisms underlying the differential responses to retinoids, although our previous research has identified specific targets in *C. albicans* [18,19]. In particular, retinoids have been suggested to interact with proteins involved in stress response and ergosterol biosynthesis, such as Hsp90 and lanosterol 14-α-demethylase [18,19]. Identifying specific genetic or biochemical pathways involved would provide a stronger mechanistic insight into retinoid activity. Future studies will, therefore, investigate the effects of these compounds on fungal membrane integrity and the induction of reactive oxygen species (ROS) production to further clarify their antifungal mechanism of action. In addition, further research is needed to evaluate potential interactions between retinoids and conventional antifungal therapies, as current limitations prevent definitive conclusions regarding synergistic or additive effects.

## 5. Conclusions

Our previous in vitro and in vivo studies have demonstrated the efficacy of retinoids against the most common fungal pathogens, such as *A. fumigatus* and *C. albicans*. Here, for the first time, the in vitro effect of retinoids against non-*albicans Candida* species, including the emerging MDR *C. auris*, was evaluated. Our results highlight that ATRA, tazarotene, and trifarotene exert an important dose-dependent and species-specific antifungal effect, particularly in reducing growth and biofilm formation in NAC species, including fluconazole-resistant *C. krusei* and *C. tropicalis*. These findings expand the known antifungal spectrum of retinoids and support their potential as promising candidates for the development of alternative or adjunctive therapeutic strategies against drug-resistant *Candida* infections. Moreover, the differential responses of *Candida* species to each retinoid may enable the use of these compounds in species-specific, tailored antifungal therapies. In conclusion, repurposing retinoids represents a compelling avenue for antifungal drug discovery. Their established clinical use, coupled with emerging antifungal properties, provides a strong rationale for further investigation into their role as antifungal agents, particularly against drug-resistant *Candida* spp. This study should be regarded as a proof of concept, highlighting promising species-specific patterns of retinoid activity that warrant further mechanistic and translational research.

## 6. Patents

The content is the object of Italian Patent Application No. 102024000028625 filed on 16 December 2024.

## Figures and Tables

**Table 1:** MIC values of NAC species. The results are the geometric means of the MIC values (µg/mL). Resistance is defined as the following MIC in micrograms per milliliter through tentative breakpoints provided by the CDC for *C. auris*: FLU ≥ 8 for *C. krusei* and *C. tropicalis*; ≥64 for *C. glabrata* and ≥32 for *C. auris.* AmB ≥ 2 for *C. auris*; VOR ≥ 2 for *C. krusei* and ≥1 for *C. tropicalis*. MICA ≥ 4 for *C. auris*; ≥0.25 for *C. glabrata* and ≥1 for *C. tropicalis* and *C. krusei*; CASPO and ANID ≥ 0.5 for *C. glabrata*; ≥1 for *C. tropicalis* and *C. krusei* [26,27].

	*C. tropicalis*	*C. auris*	*C. glabrata*	*C. krusei*
Amphotericin B	0.50	1.00	0.50	0.50
Fluconazole	8.00	8.00	0.50	8.00
Isavuconazole	0.015	0.12	0.06	0.015
Itraconazole	0.03	0.12	0.06	0.03
Posaconazole	0.015	0.06	0.03	0.015
Voriconazole	0.015	0.06	0.03	0.015
Micafungin	0.03	0.06	0.06	0.06
Anidulafungin	0.06	0.07	0.06	0.06
Caspofungin	0.06	0.12	0.06	0.06

## Data Availability

The original contributions presented in the study are included in the article/Supplementary Materials; further inquiries can be directed to the corresponding author.

## Supplementary Materials

The following supporting information can be downloaded at: https://www.mdpi.com/article/10.3390/microorganisms14040759/s1, Figure S1. Growth inhibitory activity of retinoids against NAC species. Data are presented as mean ± SD of three separate experiments, each performed in triplicate, and expressed as a percentage of growth inhibition vs. control for (A) *C. auris* CDC11930, (B) *C. tropicalis*, (C) *C. glabrata*, and (D) *C. krusei*. Two-way ANOVA, * *p* < 0.05; ** *p* < 0.01; *** *p* < 0.001; and **** *p* < 0.0001. Figure S2. AmB (2.2–0.14 mM) was used as a positive control drug for NAC growth. The absorbance intensity was measured using a spectrophotometer plate reader at 610. Data are presented as mean ± SD of three separate experiments carried out in triplicate. One-way ANOVA, * *p* < 0.05; ** *p* < 0.01; *** *p* < 0.001; and **** *p* < 0.0001. AmB, Amphotericin B. Figure S3. Adhesion inhibitory activity of retinoids against NAC species. Data are the mean ± SD of three separate experiments, each performed in triplicate, and expressed as adhesion inhibition vs. control for (A) *C. auris* CDC11903, (B) *C. tropicalis*, (C) *C. glabrata*, and (D) *C. krusei*. Two-way ANOVA, * *p* < 0.05; ** *p* < 0.01; *** *p* < 0.001; and **** *p* < 0.0001. Figure S4. Biofilm biomass inhibitory activity of retinoids against NAC species. Results are the mean ± SD of three independent experiments, each performed in triplicate, and expressed as biofilm biomass inhibition vs. control for (A) *C. auris* CDC11903, (B) *C. tropicalis*, (C) *C. glabrata*, and (D) *C. krusei*. Two-way ANOVA, * *p* < 0.05; ** *p* < 0.01; *** *p* < 0.001; and **** *p* < 0.0001. Figure S5. Biofilm metabolic inhibitory activity of retinoids against NAC species. Data are the mean ± SD of three separate experiments, each performed in triplicate, and expressed as biofilm metabolic inhibition vs. control for (A) *C. auris* CDC11930, (B) *C. tropicalis*, (C) *C. glabrata*, and (D) *C. krusei*. Two-way ANOVA, * *p* < 0.05; ** *p* < 0.01; *** *p* < 0.001; and **** *p* < 0.0001. Figure S6. AmB (2.2–0.14 mM) was used as a positive control drug for NAC biofilm biomass. Absorbance of the crystal violet dye was recorded at 595 nm using a spectrophotometer plate reader. Values are expressed as mean ± SD from three separate experiments, each conducted in triplicate. One-way ANOVA, * *p* < 0.05; ** *p* < 0.01; *** *p* < 0.001; and **** *p* < 0.0001. AmB, Amphotericin B. Figure S7. AmB (2.2–0.14 mM) was used as a positive control drug for NAC biofilm metabolic activity. The absorbance of the crystal violet dye was determined at 490 nm using a spectrophotometric plate reader. Data are presented as the mean ± SD from three independent experiments, each performed in triplicate. One-way ANOVA, * *p* < 0.05; ** *p* < 0.01; *** *p* < 0.001; and **** *p* < 0.0001. AmB, Amphotericin B.

## Abbreviations

The following abbreviations are used in this manuscript:

ATRA	All-trans retinoic acid
CLSI	Clinical and Laboratory Standards Institute
CYP51	Lanosterol 14α-demethylase
CV	Crystal violet
DMSO	Dimethyl sulfoxide
ELISA	Enzyme-linked immunosorbent assay
FCS	Fetal calf serum
FLU	Fluconazole
GM	Geometric means
Hsp90	Heat shock protein 90
MDR	Multidrug-resistant
NAC	Non-*albicans Candida*
RAR-γ	Retinoic acid receptor-γ
ROS	Reactive oxygen species
SDA	Sabouraud dextrose agar
SYO	Sensitrite YeastOne
XTT	2,3-Bis-(2-Methoxy-4-Nitro-5-Sulfophenyl)-2H-Tetrazolium-5-Carboxanilide
