# Supplementary material for: Potential Antifungal Activity of Retinoids Against Non-albicans Candida Species
Source: Microorganisms. 2026 Mar 27;14(4):759. doi: 10.3390/microorganisms14040759 (PMC13118457; doi:10.3390/microorganisms14040759)
Supplement: Supplementary file 1 [file microorganisms-14-00759-s001.zip › microorganisms-4201613-supplementary.pdf]

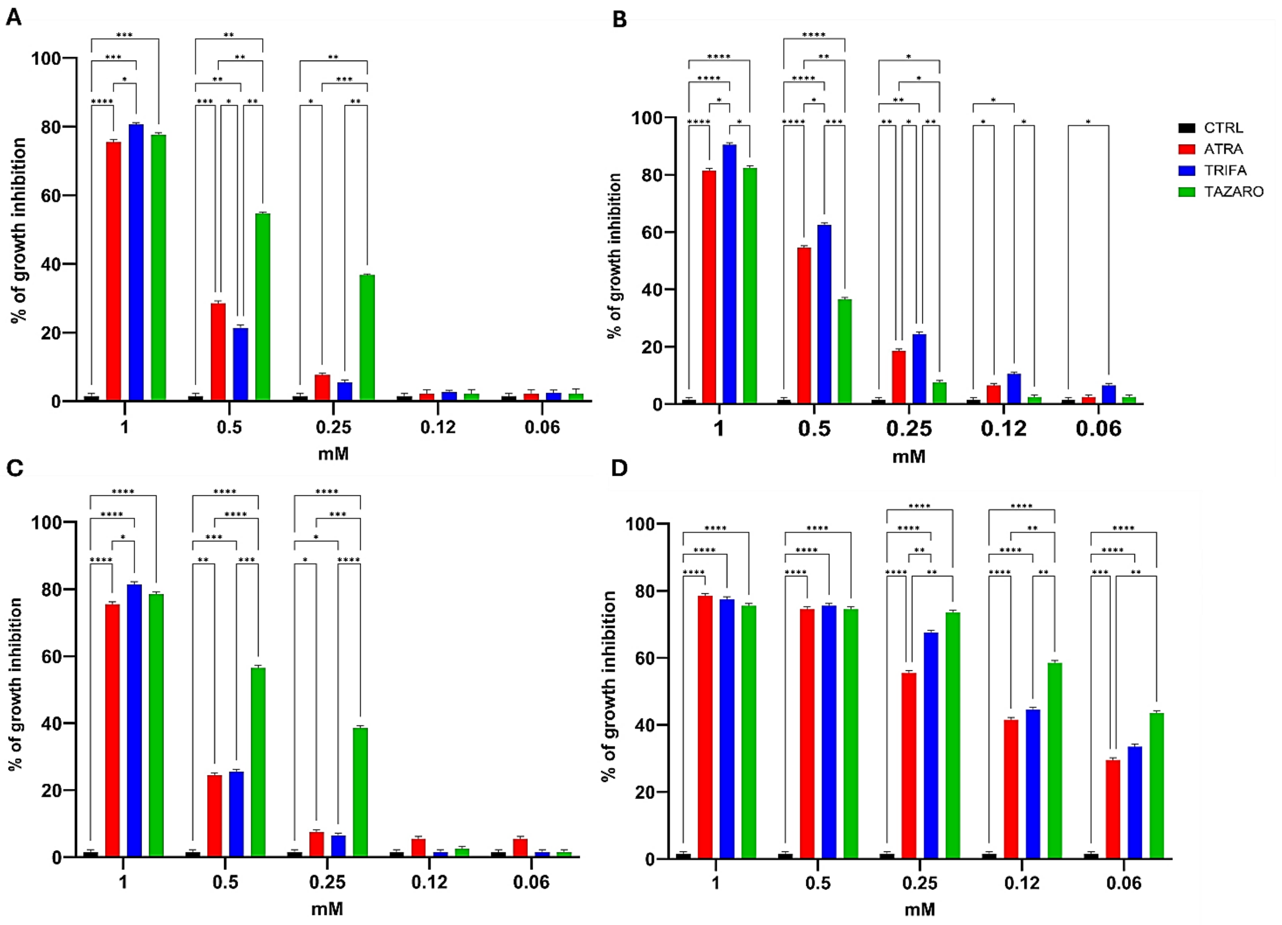

Figure S1. Growth inhibitory activity of retinoids against NAC species. Data are presented as mean ± SD of three separate experiments, each performed in triplicate, and expressed as a percentage of growth inhibition *vs*. control for (**A**) *C. auris* CDC11930, (**B**) *C. tropicalis*, (**C**) *C. glabrata* and (**D**) *C. krusei*. Two-way ANOVA, *p<0.05; **p<0.01; ***p<0.001; ****p<0.0001.

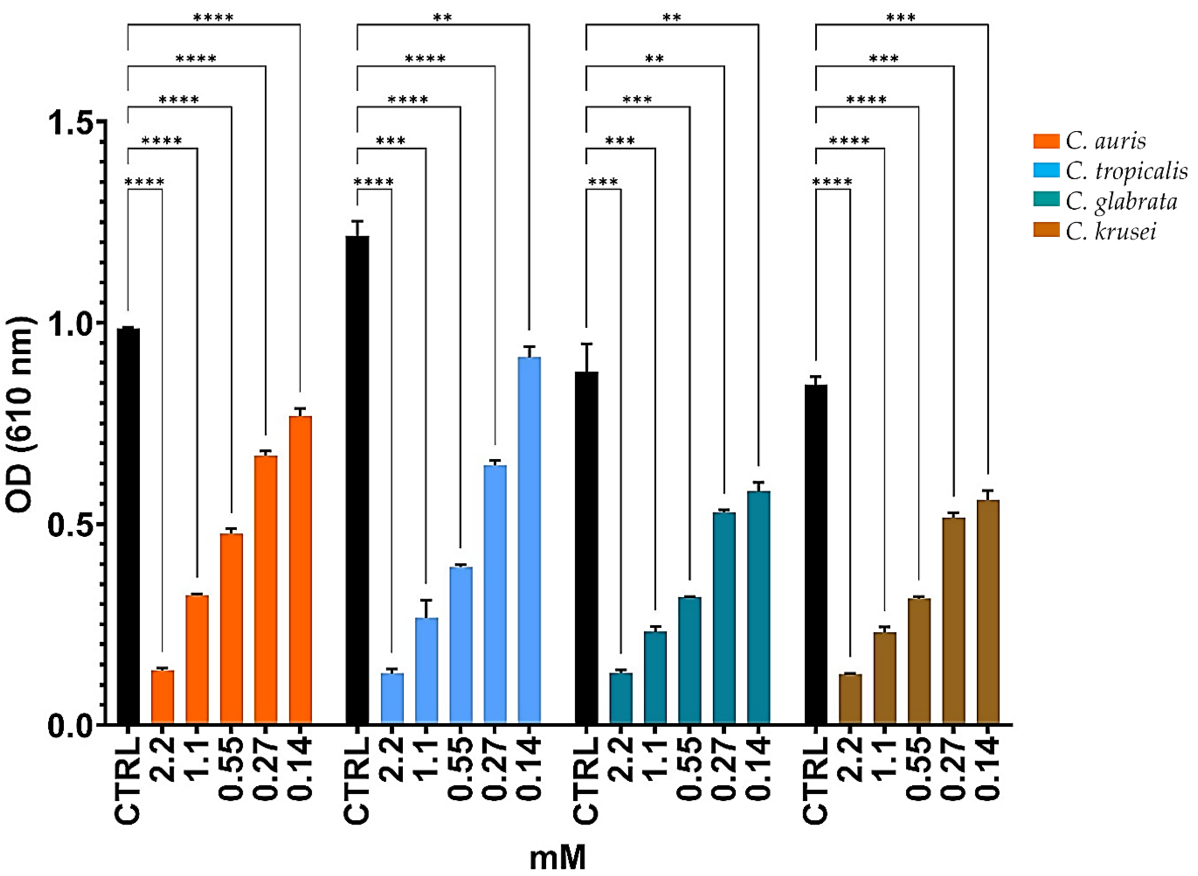

Figure S2. AmB (2.2–0.14 mM) was used as positive control drug for NAC growth. The absorbance intensity was measured using a spectrophotometer plate reader at 610. Data are presented as mean ± SD of three separate experiments carried out in triplicate. One-way ANOVA, **p*<0.05; ** *p*<0.01; *** *p*<0.001; *****p*<0.0001. AmB, Amphotericin B.

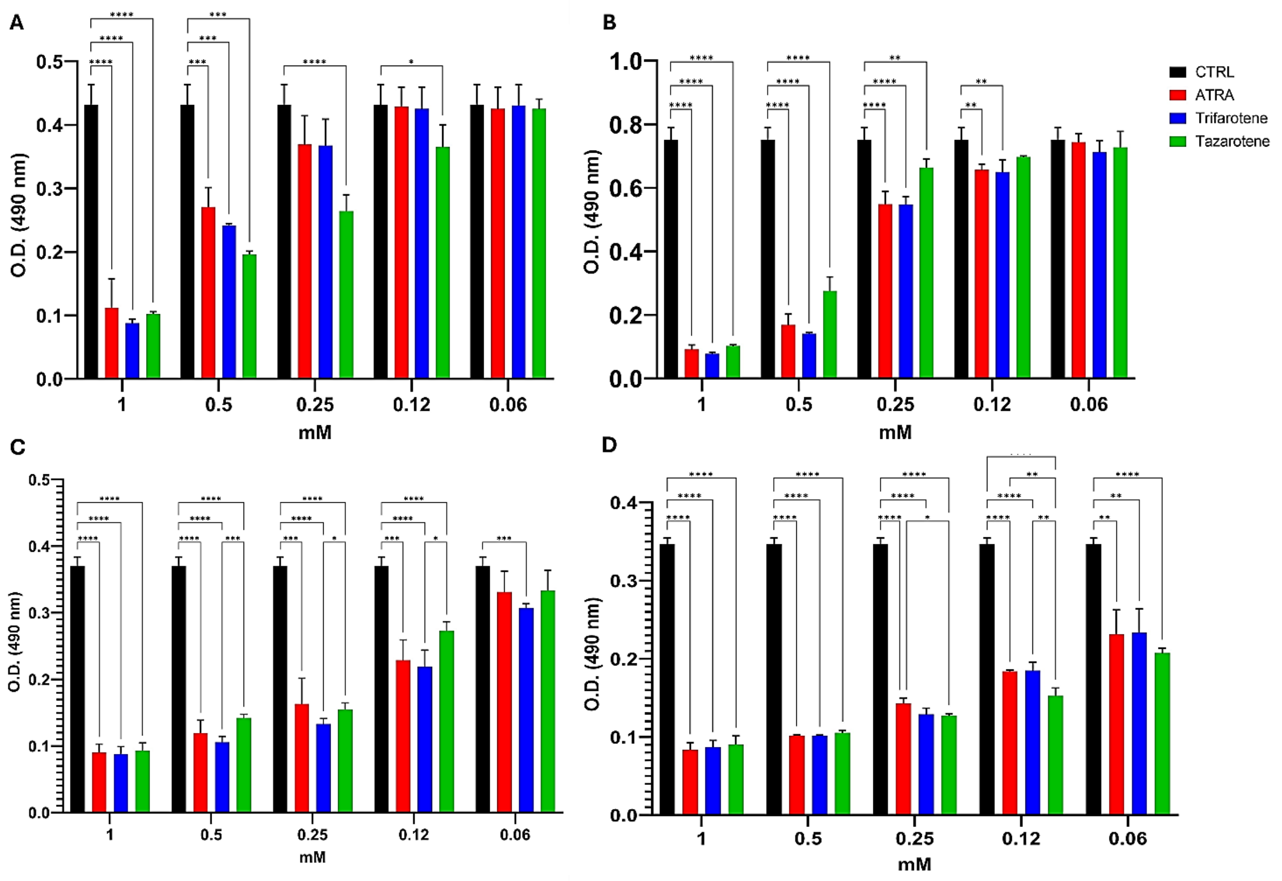

Figure S3. Adhesion inhibitory activity of retinoids against NAC species. Data are the mean ± SD of three separate experiments, each performed in triplicate, and expressed as adhesion inhibition *vs*. control for (**A**) *C. auris* CDC11930, (**B**) *C. tropicalis*, (**C**) *C. glabrata* and (**D**) *C. krusei*. Two-way ANOVA, **p*<0.05; ***p*<0.01; ****p*<0.001; *****p*<0.0001.

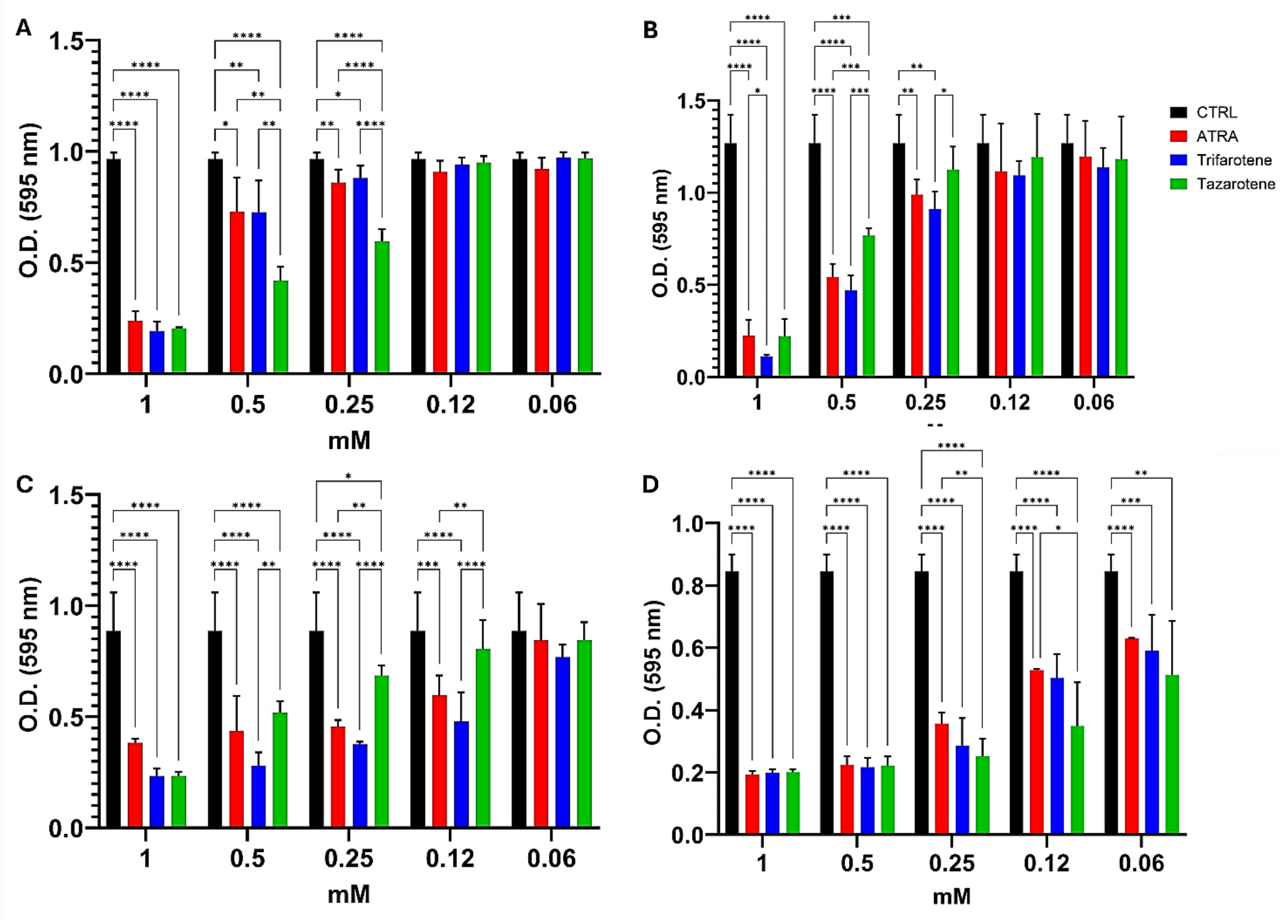

Figure S4. Biofilm biomass inhibitory activity of retinoids against NAC species. Results are the mean ± SD of three independent experiments, each performed in triplicate, and expressed as biofilm biomass inhibition *vs*. control for (**A**) *C. auris* CDC11930, (**B**) *C. tropicalis*, (**C**) *C. glabrata* and (**D**) *C. krusei*. Two-way ANOVA, *p<0.05; **p<0.01; ***p<0.001; ****p<0.0001.

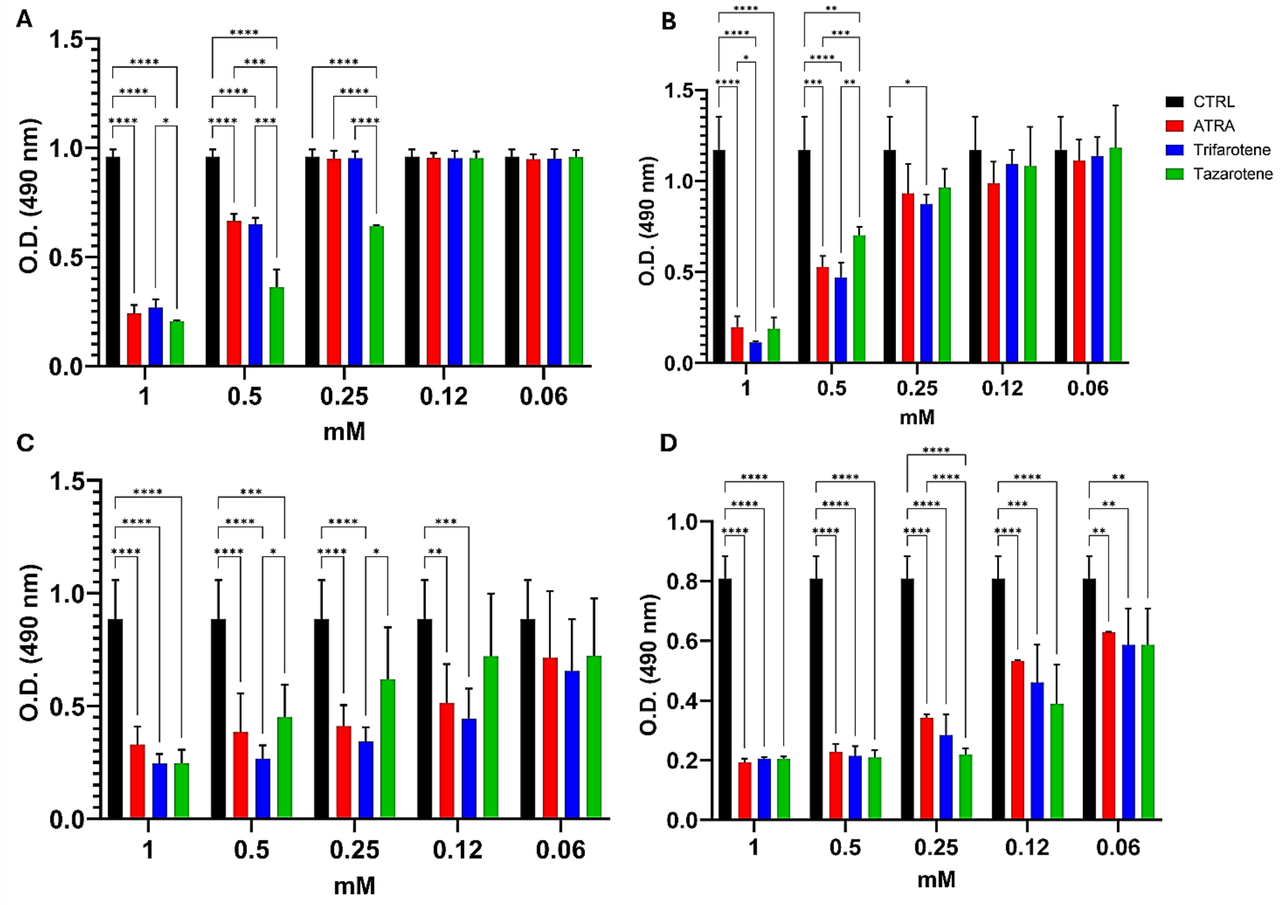

Figure S5. Biofilm metabolic inhibitory activity of retinoids against NAC species. Data are the mean ± SD of three separate experiments, each performed in triplicate, and expressed as biofilm metabolic inhibition *vs*. control for (**A**) *C. auris* CDC11930, (**B**) *C. tropicalis*, (**C**) *C. glabrata* and (**D**) *C. krusei*. Two-way ANOVA, **p*<0.05; ***p*<0.01; ****p*<0.001; *****p*<0.0001.

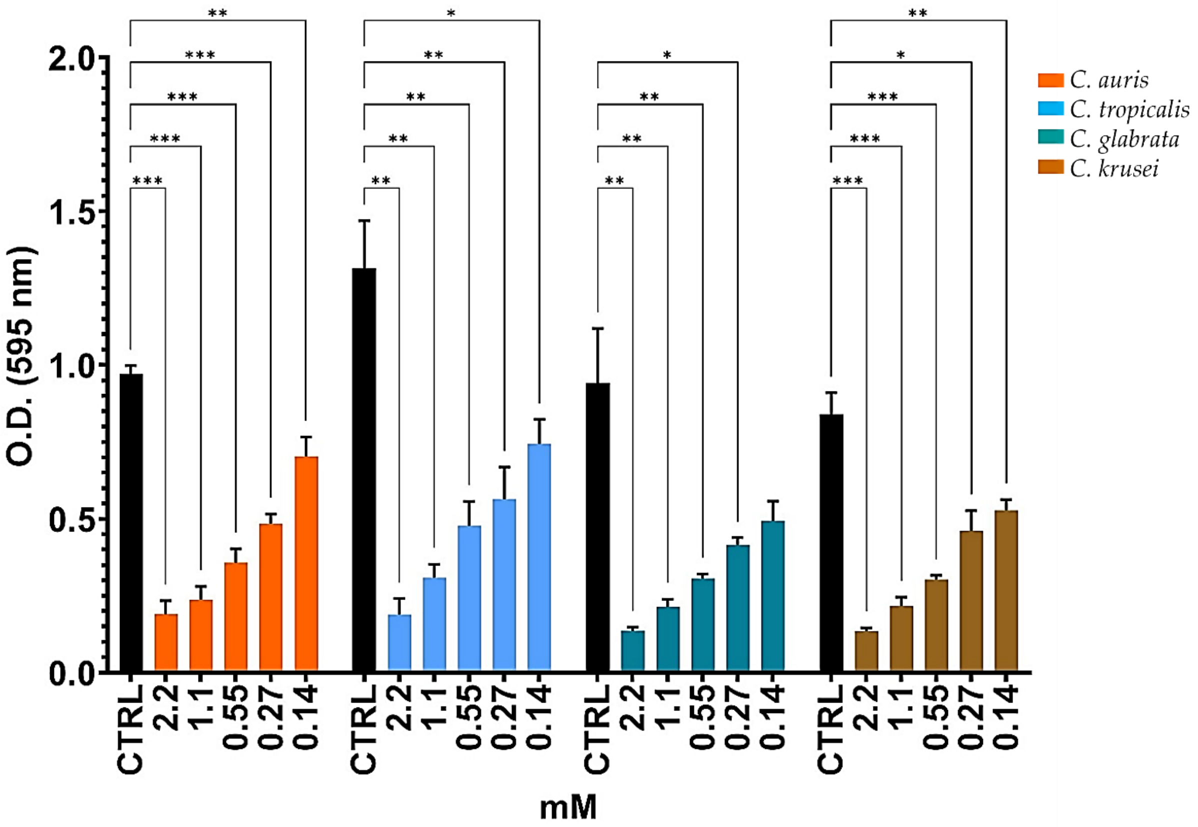

Figure S6. AmB (2.2–0.14 mM) was used as positive control drug for NAC biofilm biomass. The absorbance intensity of the crystal violet dye was measured using a spectrophotometer plate reader at 595 nm. Absorbance of the crystal violet dye was recorded at 595 nm using a spectrophotometer plate reader. Values are expressed as mean ± SD from three separate experiments, each conducted in triplicate. One-way ANOVA, *$p<0.05$; ** $p<0.01$; *** $p<0.001$; ****$p<0.0001$. AmB, Amphotericin B.

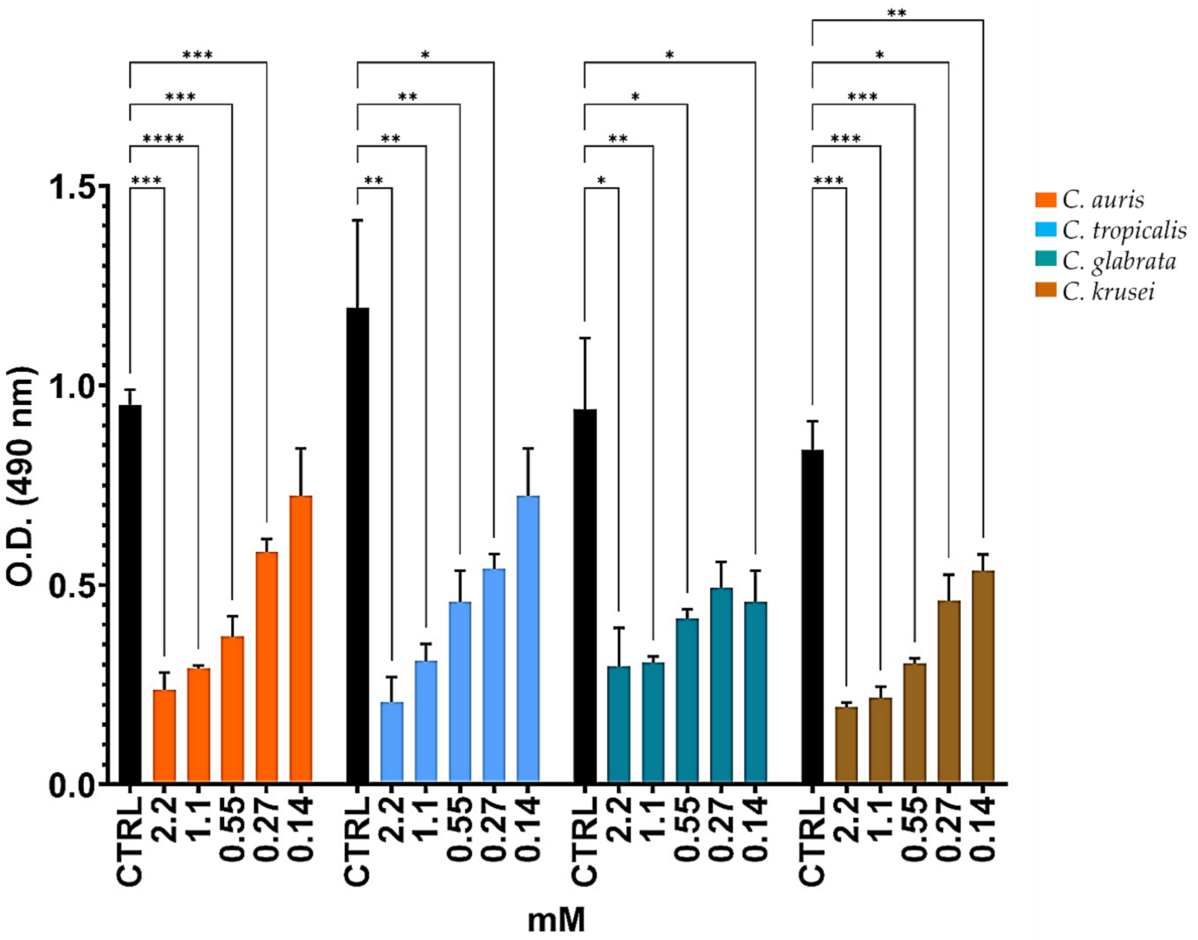

Figure S7. AmB (2.2–0.14 mM) was used as positive control drug for NAC biofilm metabolic activity. The absorbance intensity of the crystal violet dye was measured using a spectrophotometer plate reader at 490 nm. The absorbance of the crystal violet dye was determined at 490 nm using a spectrophotometric plate reader. Data are presented as the mean ± SD from three independent experiments, each performed in triplicate. One-way ANOVA, *$p<0.05$; ** $p<0.01$; *** $p<0.001$; ****$p<0.0001$. AmB, Amphotericin B.
